# Prostaglandin E2 alters Wnt-dependent migration and proliferation in neuroectodermal stem cells: implications for autism spectrum disorders

**DOI:** 10.1186/1478-811X-12-19

**Published:** 2014-03-23

**Authors:** Christine T Wong, Eizaaz Ahmad, Hongyan Li, Dorota A Crawford

**Affiliations:** 1 School of Kinesiology and Health Science, York University, 4700 Keele Street, Toronto, Ontario M3J 1P3, Canada; 2 Neuroscience Graduate Diploma Program, York University, 4700 Keele Street, Toronto, Ontario M3J 1P3, Canada; 3 Department of Biology, Faculty of Health, York University, 4700 Keele Street, Toronto, Ontario M3J 1P3, Canada

**Keywords:** Prostaglandin E2, Wnt signalling, Neuroectodermal stem cells, Cell motility, Proliferation, Autism

## Abstract

Prostaglandin E2 (PGE_2_) is a natural lipid-derived molecule that is involved in important physiological functions. Abnormal PGE_2_ signalling has been associated with pathologies of the nervous system. Previous studies provide evidence for the interaction of PGE_2_ and canonical Wnt signalling pathways in non-neuronal cells. Since the Wnt pathway is crucial in the development and organization of the brain, the main goal of this study is to determine whether collaboration between these pathways exists in neuronal cell types. We report that PGE_2_ interacts with canonical Wnt signalling through PKA and PI-3K in neuroectodermal (NE-4C) stem cells. We used time-lapse microscopy to determine that PGE_2_ increases the final distance from origin, path length travelled, and the average speed of migration in Wnt-activated cells. Furthermore, PGE_2_ alters distinct cellular phenotypes that are characteristic of Wnt-induced NE-4C cells, which corresponds to the modified splitting behaviour of the cells. We also found that in Wnt-induced cells the level of β-catenin protein was increased and the expression levels of Wnt-target genes (*Ctnnb1*, *Ptgs2*, *Ccnd1*, *Mmp9*) was significantly upregulated in response to PGE_2_ treatment. This confirms that PGE_2_ activated the canonical Wnt signalling pathway. Furthermore, the upregulated genes have been previously associated with ASD. Our findings show, for the first time, evidence for cross-talk between PGE_2_ and Wnt signalling in neuronal cells, where PKA and PI-3K might act as mediators between the two pathways. Given the importance of PGE_2_ and Wnt signalling in prenatal development of the nervous system, our study provides insight into how interaction between these two pathways may influence neurodevelopment.

## Background

The plasma membrane phospholipids play a fundamental role in the nervous system and act as a reservoir for second messenger molecules important for the development and normal functioning of the brain. Prostaglandin E2 (PGE_2_) is a bioactive fatty acid that is derived from arachidonic acid, a major structural component of plasma membrane phospholipids, through the enzymatic metabolism of cyclooxygenases −1 and −2 (COX-1,-2) and then prostaglandin synthases [1]. Extracellular stimuli such as immunological and infectious agents [2-4], environmental toxins such as mercury and lead [5], and exposure to drugs including misoprostol and valproic acid [6] can trigger the local production of PGE_2_ via specific biosynthetic pathways, resulting in altered cell signal transmission that modulates biological functions such as sleep, fever, inflammation, and pain [7].

The diverse action of PGE_2_ is achieved through the activation of 4 different G-protein coupled E-prostanoid receptors (EP1 through 4) [8,9]. The divergent role of PGE_2_ is amplified by the variety of different kinase-mediated signalling cascades that can be activated through its EP receptors, such as the protein kinase A (PKA), phosphatidylinositide 3-kinases (PI-3K), and protein kinase C (PKC) pathways [10].

During the early stages of pregnancy, there are elevated levels of COX-2 and PGE synthases, enzymes responsible for the production of PGE_2_, which is indicative of the involvement of PGE_2_ in prenatal development [11]. We have previously shown that the expression profiles of EP receptors during mouse embryonic development changes depending on the embryonic stage, with EP receptor expression highest during E7 (Embryonic day 7) and E15, which corresponds to peak periods of neurogenesis [12]. It has been shown that PGE_2_ plays a regulatory role in membrane excitability and synaptic transmission in neurons [13]. PGE_2_ increases the dendritic length and complexity of Purkinje neurons, and can also alter neuronal firing activity in the developing brain [14]. PGE_2_ is involved in synaptic plasticity and neuroprotection [15], and can also be involved in neuronal cell death and apoptosis [16,17]. Prostaglandins have also been reported to induce the differentiation of neuronal cells [18]. Moreover, the inhibition of COX-2, can suppress neurogenesis and proliferation of neural progenitor cells [19]. These studies show the important role PGE_2_ can play during normal development of the nervous system. Furthermore, previous research found that PGE_2_ can exert various effects on cell development, proliferation, and migration in a diversity of cell lines. It has been shown that PGE_2_ stimulates cell growth and motility in osteoblasts [20], prostate cancer cells [21], and pancreatic stellate cells [22]. The migration of vascular smooth muscle cells [23], intestinal subepithelial myofibroblasts [24], dendritic cells [25], hepatocellular carcinoma cells [26], and mesangial cells [27] can all be regulated by PGE_2_. However, the effects of PGE_2_ on neural stem cell behaviour and movement are not well characterized. Our previous studies provide some insight into the molecular mechanisms of abnormal PGE_2_ signalling on neuronal cells. We have found that exposure to PGE_2_ results in the retraction of neurites and the elevation of calcium amplitude fluctuations in growth cones of differentiated Neuro-2A cells [12,28].

Abnormal fatty acid metabolism through the PGE_2_ pathway may contribute to the pathology of neurodevelopmental disorders such as Autism Spectrum Disorders (ASD) [29]. Abnormal levels of PGE_2_ and other fatty acid metabolites have been identified as potential biomarkers for ASD [30]. PGE_2_ can act as an endogenous modulator for cerebellar development in the rat brain affecting social interaction and sensory behaviour, which are characteristic behaviours altered in ASD [31]. A clinical study showed that maternal exposure to the drug misoprostol (prostaglandin E analogue), has been associated with the development of Moebius syndrome and autistic-like symptoms [32-34].

Current literature also provides evidence that PGE_2_ signalling interacts with another crucial developmental pathway, the canonical Wnt (*wingless-related MMTV integration site*) signalling pathway in various non-neuronal cells [35] such as osteocytes [36], prostate and colon cancer cells [37], hematopoietic stem cells [38], and mesenchymal stem cells [39]. Wnt signalling is tightly regulated in early development and is required for the formation of the nervous system [40]. The canonical Wnt signalling pathway is composed of a network of proteins that modify cell communication and interactions with other cells. Wnt proteins bind to cell surface Frizzled (FZD) receptors, where the signal is then transduced to β-catenin, activating the transcription of Wnt target genes. The Wnt molecules are vital to embryonic development since they can moderate cell proliferation and differentiation by participating in the determination of cell fates [41]. Previous literature shows that convergence of PGE_2_-dependent signalling with the canonical Wnt pathway can occur at the level of β-catenin through EP1-4 receptors, including the association of the G_αs_ subunit with Axin, the stimulation of the cAMP/PKA pathway, or the phosphorylation of GSK-3β by PI-3K [42]. However, the interaction of PGE_2_ and Wnt signalling is not well-characterized in the nervous system. To activate and study canonical Wnt signalling in an in vitro model system, Wnt Agonist (WntA), 2-amino-4-[3,4-(methylenedioxy)benzylamino]-6-(3-methoxyphenyl) pyrimidine, can serve as a useful reagent. WntA is a cell-permeable pyrimidine compound that mimics the effects of Wnt by functioning through the canonical pathway via upregulating TCF-activity without inhibiting the activity of GSK-3β [43]. This is important because GSK-3β plays a regulatory role in many signalling pathways other than Wnt so an agonist that blocks GSK-3β could have disparate effects in cellular models.

This study investigates the effects of PGE_2_ interaction with the Wnt signalling pathway on the behaviour of murine neuroectodermal (NE-4C) stem cells. We demonstrate that PGE_2_ modifies canonical Wnt signalling in NE-4C stem cells by altering components of cell motility such as final distance travelled, path length travelled, average speed of migration, as well as cell splitting behaviour. We also reveal that PGE_2_ can alter the protein expression of non-phospho (active) β-catenin (Ser33/37/Thr41), as well as the expression of specific Wnt-target genes. Interestingly, the genes implicated in our study have been previously associated with ASD. To our knowledge, we show for the first time, that PGE_2_ signalling interacts with the Wnt pathway in neural stem cells to affect cell behaviour and gene transcription. Our study furthers our understanding of the possible mechanisms by which intracellular cross-talk between PGE_2_ and Wnt signalling may contribute to cell migration and proliferation during prenatal development of the nervous system.

## Results

### Expression of EP1-4 receptors in NE-4C cells

**Figure 1:**
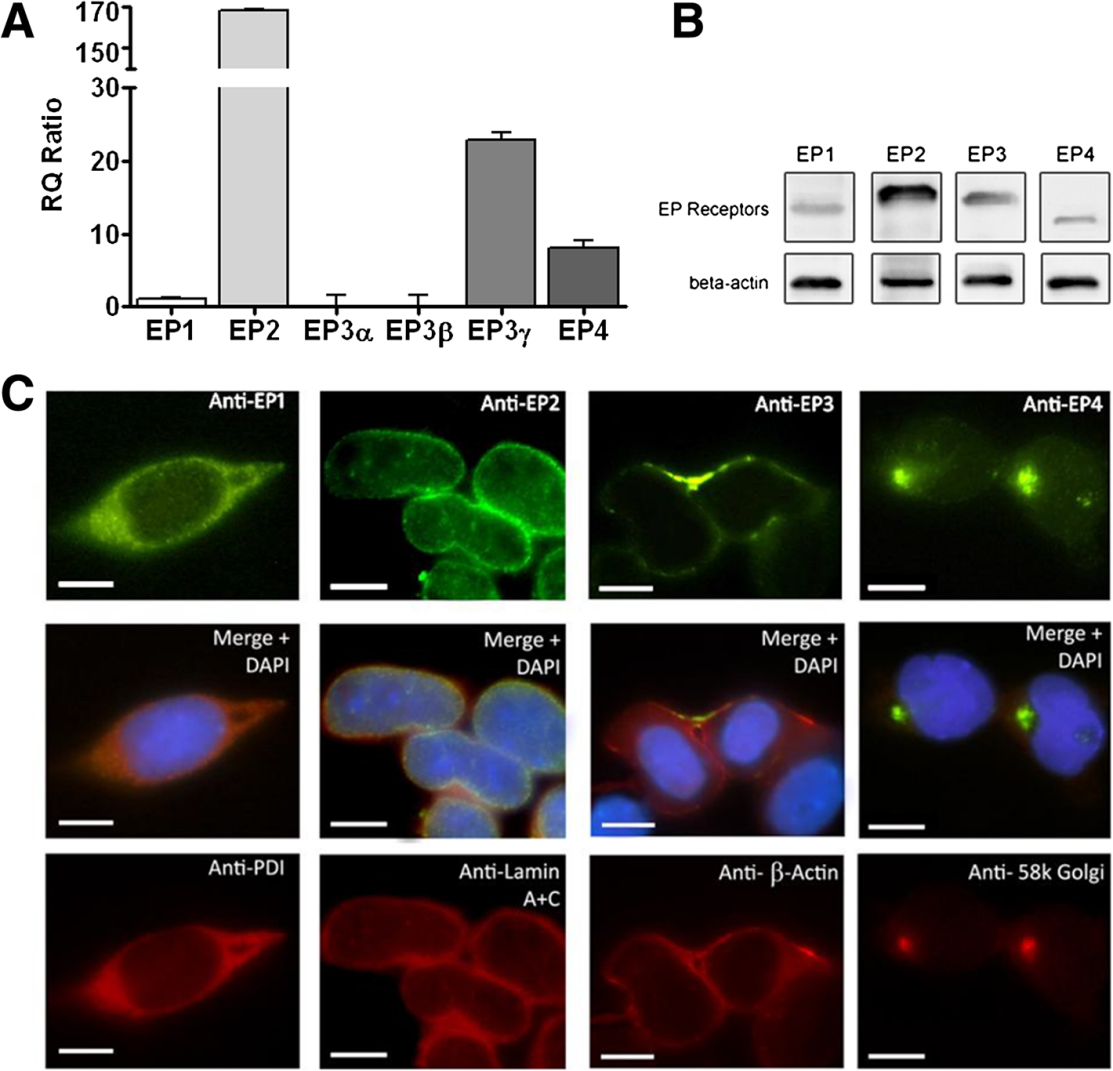
**Expression of EP receptors’ mRNA and protein in NE-4C cells. (A)** Real-time PCR was used to determine the RQ value for EP1, EP2, EP3α, EP3β, EP3γ and EP4 receptors, which was found to be 2, 16, 1, 2, 46 and 46 respectively. The error bars represent + SEM. **(B)** Western blot analysis of the EP1, EP2, EP3 and EP4 receptors expression (65, 68, 62 and 53 kDa, respectively). β-Actin was used to indicate equal loading. **(C)** Immunocytochemistry revealed the subcellular localization of EP1-4 receptors with specific organelles visualized through the use of anti-PDI endoplasmic reticulum marker, anti-Lamin A + C nuclear envelope marker, *β*-Actin cell membrane marker, and anti-58 K Golgi marker. The scale bar represents 10 μm.

To determine whether NE-4C cells endogenously express the receptors of PGE_2_, we performed real-time quantitative PCR assay, Western blot analysis, and immunocytochemistry. Our results show that in NE-4C cells, EP2 had the highest mRNA expression followed by EP3γ and EP4 receptors. Endogenous EP1 and EP3β receptor expression was considerably low in NE-4C cells, while the EP3α transcript level was nearly absent and may be considered negligible. The relative quantity (RQ = 1) values of EP1, EP2, EP3α, EP3β, EP3γ, and EP4 transcripts expression were 3, 542, 0, 1, 391, and 15, respectively (Figure 1A). Western blot results confirm the expression of all four EP receptors in NE-4C cells (Figure 1B). The localization of the EP receptors in NE-4C cells was also detected with immunocytochemistry using EP1-4 specific antibodies along with antibodies against various cellular organelles including the nuclear envelope, Golgi apparatus, the endoplasmic reticulum, and β-Actin (Figure 1C). Our results show that EP1 receptors were localized in the ER membrane, EP2 receptors were uniformly expressed around the nucleus and co-localized with the nuclear envelope marker, EP3 receptors were located at the plasma membrane, and EP4 receptors at the Golgi apparatus. Hence, NE-4C cells can act as an appropriate experimental model to study PGE_2_ signalling.

### Prostaglandin E_2_ increases the cell motility of Wnt-induced NE-4C cell migration

The effect of PGE_2_ on Wnt-dependent migration of NE-4C cells was determined using Nikon Eclipse Ti-E microscope with NIS Elements time-lapse tracking software over a 24 hour period. Final distance, path length, and average speed were quantified after exposure to 1 μM PGE_2_, 2 μM Wnt Agonist (WntA), or 2 μM WntA with the addition of 1 μM PGE_2_. The *final distance* was defined as the distance between the initial and final positions of the cell, represented as a straight line distance. The *path length* was the total distance travelled from the initial to the final cell position. The *average speed* of a cell was calculated by dividing the total distance travelled by the time it took to travel between the two positions.

**Figure 2:**
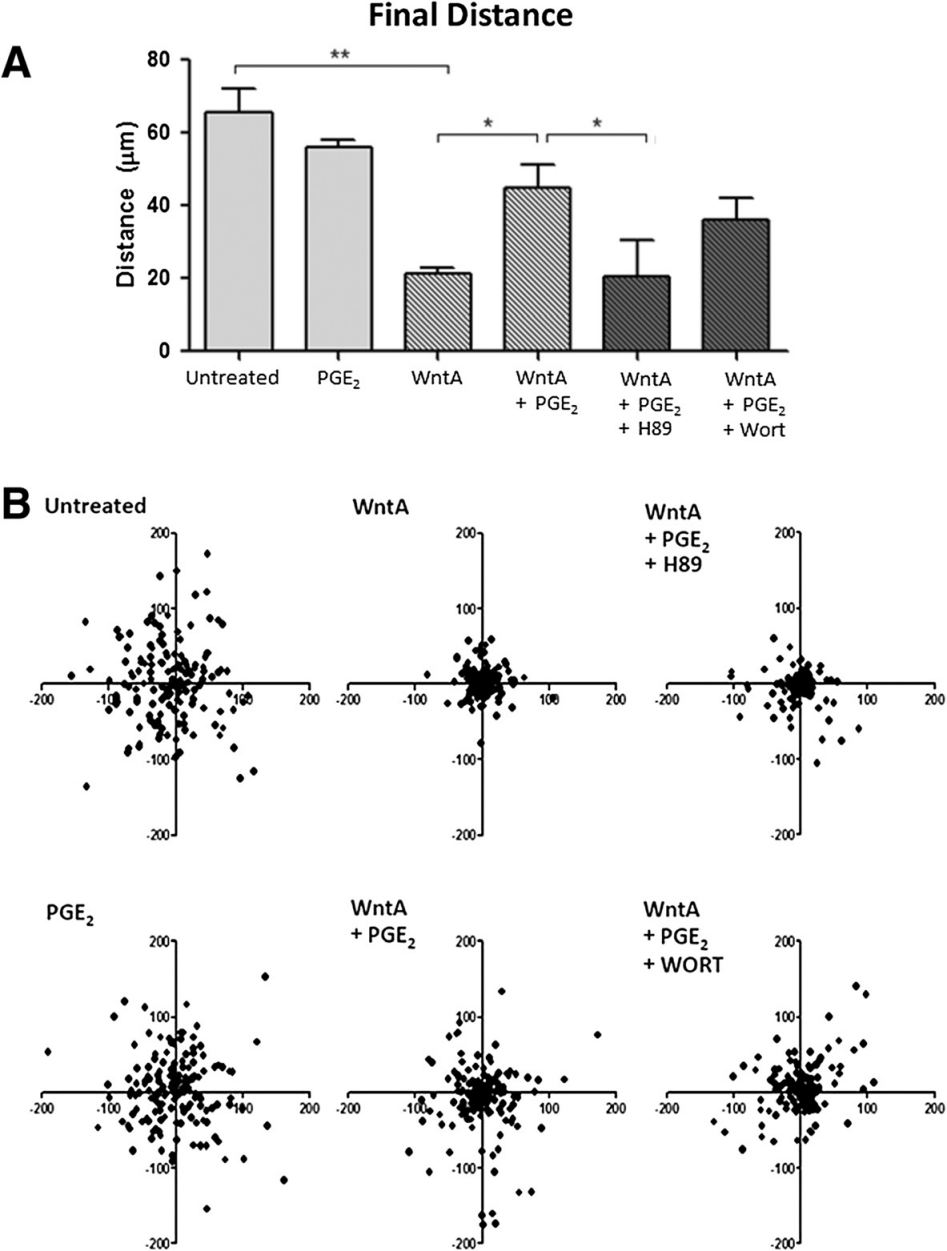
**PGE**_**2**_**-dependent effect on final distance travelled from origin. (A)** Final distance from origin was 65.6, 56.2, 21.3, 45.0 μm, respectively. The error bars represent + SEM and values were considered significant at *p < 0.05, **p < 0.01. **(B)** The Dispersion XY position plots illustrate the effect of PGE_2_ on Wnt-induced behaviour, where addition of PGE_2_ to Wnt-activated cells increased the final distance. Addition of H89 (PKA blocker) and Wort (PI-3K) blocker reduces the effect PGE_2_. Measurements represent an average of 150 cells from three independent experiments (*N* = 3).

The results show that untreated NE-4C cells moved an average final distance of 65.6 μm following a 24 hour period (Figure 2A). The addition of PGE_2_ to the cells resulted in a final distance of 56.2 μm which was not significantly different from the untreated control (65.6 μm). WntA only treatment resulted in a significant decrease in final distance of 21.3 μm (*p* = 0.00242) when compared to the control. The addition of PGE_2_ to WntA-treated cells resulted in a final distance of 45.0 μm, which is an increase by 23.6 μm (*p* = 0.04371), as compared to WntA only-treated cells. It represents a 211% increase from the WntA-regulated movement. Visualization of final distance through dispersion XY position plots clearly illustrates that PGE_2_ signalling restores the Wnt-regulated suppression of cell movement (Figure 2B, *WntA + PGE2*).

**Figure 3:**
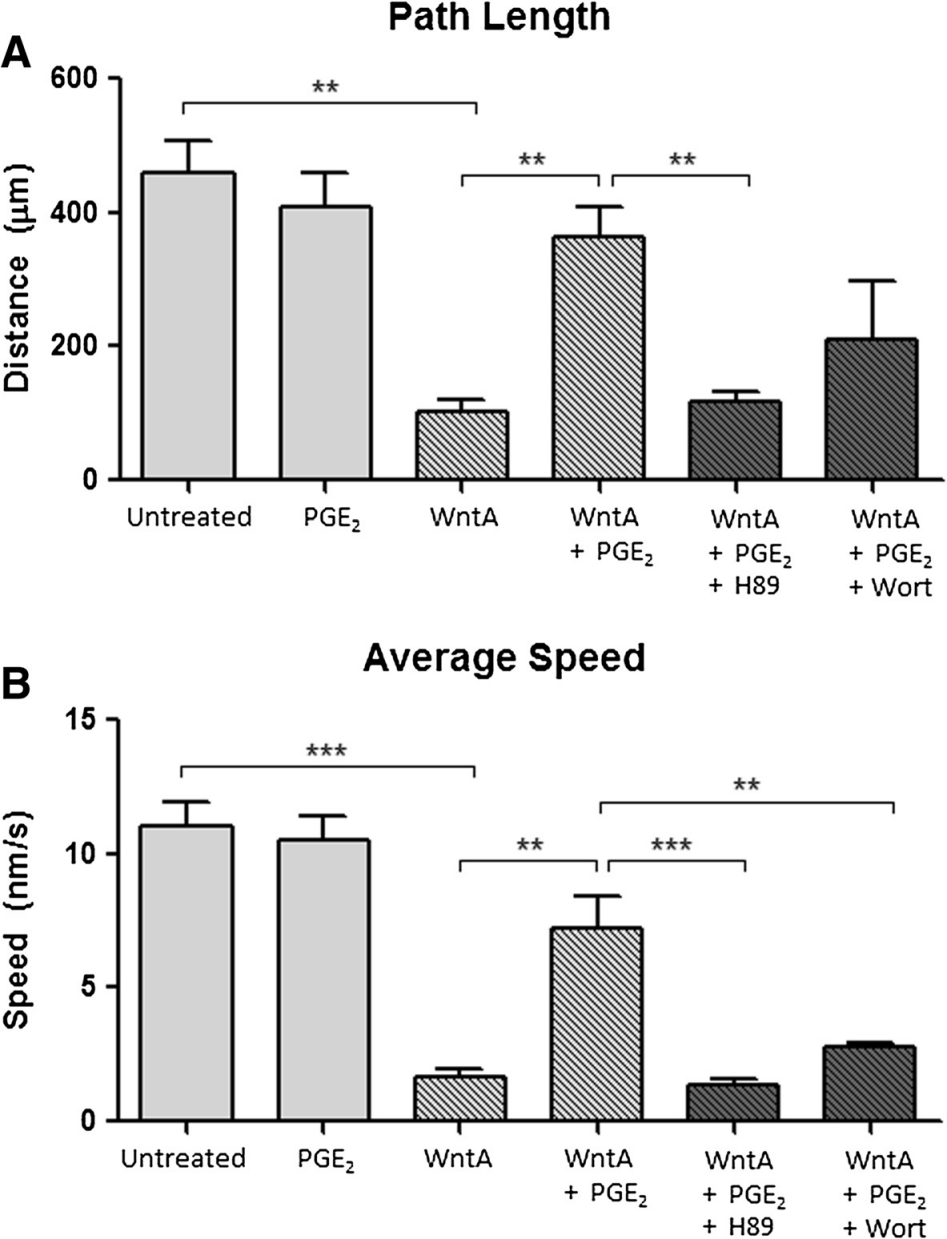
**PGE**_**2**_**-dependent effect on path length and average speed. (A)** Path length travelled was 459, 409, 103, 362 μm, respectively. **(B)** Average speed of migration was 11.0, 10.5, 1.7, 7.2 nm/s, respectively. The error bars represent + SEM, **p < 0.01, ***p < 0.001. Results represent an average of 150 cells from three independent experiments (*N* = 3).

The quantification of path length (Figure 3A) revealed the same pattern. The path length of untreated cells was 458.9 μm. As compared to untreated cells, PGE_2_ only treatment did not result in a significant change (408.6 μm), but WntA treatment significantly decreased the path length to 103.3 μm (*p* = 0.00189). Addition of PGE_2_ to WntA-treated cells led to a path length of 362.1 μm. This is a 350% increase (*p* = 0.00928) compared to WntA only-treated cells.

Quantification of average speed showed that PGE_2_ treated cells travelled at a speed of 10.5 nm/s, which was not significantly different from untreated NE-4C cells that moved at a speed of 11.0 nm/s (Figure 3B). WntA only treatment resulted in a decreased average cell speed of 1.65 nm/s (*p* = 0.00065). Addition of PGE_2_ to WntA-treated cells resulted in an average speed of 7.34 nm/s. This suggests that addition of PGE_2_ elevated the average speed by 439%; an increase of 5.59 nm/s (*p* = 0.00946) when compared to WntA only-treatment.

In summary, administration of PGE_2_ treatment leads to significant changes in WntA-regulated cell behaviours such as final distance, path length, and average speed. PGE_2_ treatment significantly restored the cell kinematic measures which were suppressed by WntA treatment.

### Prostaglandin E_2_ modulates Wnt-induced cell behaviour through PKA and PI-3K kinases

Previous studies in embryonic kidney and colon cancer cells determined that the convergence of PGE_2_ signalling on the Wnt pathway occurred through the activation of PKA or PI-3K [44-46]. To determine whether PGE_2_ treatment alters Wnt-induced cell migration behaviour via these kinases in NE-4C cells, we used dihydrochloride hydrate (H89) to block PKA and Wortmannin (Wort) to block PI-3K. Our results show a trend across final distance, path length, and average speed (Figures 2 and 3). With the addition of H89 to WntA + PGE_2_ treated cells, all cell motility measures significantly decreased compared to the WntA + PGE_2_ treated cells, resulting in movement profiles that were not statistically different from the WntA-only condition. Specifically, H89-treated cells travelled a final distance of 20.32 μm from the origin (*p* = 0.02477), path length of 116.01 μm (*p* = 0.00567), and at an average speed of 1.37 nm/s (*p* = 0.00073) (Figure 2A and 2B).

With the addition of Wort to WntA + PGE_2_ treated cells, there was a decreasing trend in final distance and path length but it was not significantly different from PGE_2_ + WntA treated cells. Only average speed significantly decreased to 2.76 nm/s (*N* = 3*; p* = 0.00422) compared to the WntA + PGE_2_ treatment. Post hoc Dunnett t-test revealed that measurements from the H89 and Wort conditions were not significantly different from the WntA-only treatment, indicating that H89 and Wort significantly diminished the effect of PGE_2_ on WntA-treated cells. This indicates that PGE_2_ likely acts through PKA and PI-3K to elicit effects on the WntA-dependent cell motility. However, it appears that H89 may have had a greater effect, suggesting that PGE_2_ may predominately act through PKA.

### Prostaglandin E_2_ alters cell proliferation behaviour of NE-4C cells induced by Wnt agonist treatment

**Figure 4:**
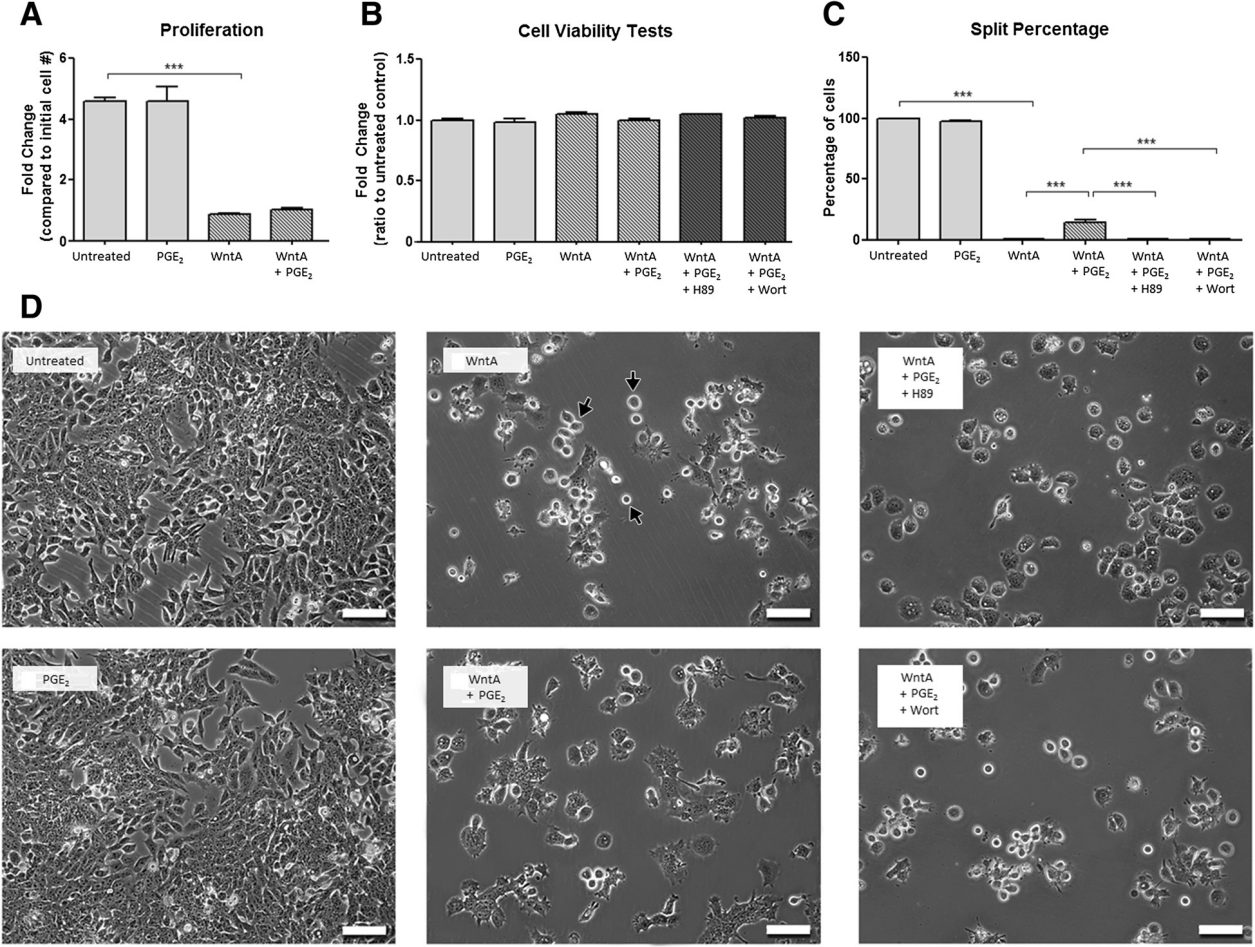
**PGE**_**2**_**-dependent effect on proliferation behaviour. (A)** Over the experimental duration of 24 hours, the number of cells changed by a fold of 4.60, 4.59, 0.86, 1.03, respectively. **(B)** Cell viability across treatment conditions was not significantly different. **(C)** Percentage of successful split ratio was 100%, 98%, 0%, 15%, 0%, and 0% respectively. The error bars represent + SEM, ***p < 0.001. Measurements represent an average of 150 cells from three independent experiments (*N* = 3). **(D)** WntA treatment resulted in an arrested state indicted by the black arrows and corresponded with a significant decrease in cell proliferation (***p < 0.001). Scale bar represents 100 μm.

Previous literature reveals that PGE_2_ may also affect cell proliferation via the Wnt signalling pathway in prostate and colon cancer cells [37] and hematopoietic [38] and mesenchymal [39] stem cells. We studied the effects of PGE_2_ on NE-4C cell proliferation using NIS Elements software. The cells were exposed to 1 μM PGE_2_, 2 μM WntA, or 2 μM WntA with the addition of 1 μM PGE_2_. Furthermore, H89 or Wort was added to PGE_2_ + WntA treated cells to determine the effective role of these kinases. The initial number of cells was compared to the final number of cells following 24 hours treatment. PGE_2_ treatment led to an increase in cell number by 4.60-fold, which was not significantly different from the untreated cells that proliferated by a 4.59 fold-increase (Figure 4A). Administration of WntA resulted in a fold-change of 0.86 (*p* < 0.001) which was significantly lower than untreated cells. Addition of PGE_2_ to WntA-treated cells (WntA + PGE_2_) resulted in a fold-change of 1.03, which was not significant from the WntA only treated condition. Although we observed lower levels of proliferation in the WntA, WntA + PGE_2_ and WntA + PGE_2_ + Blocker conditions, we confirmed no change in cell viability between the conditions tested (Figure 4B).

However, we observed distinct differences in cell phenotype between the WntA, WntA + PGE_2_ and WntA + PGE_2_ with H89 or Wort treatment. A majority of the cells treated with WntA adopted a shiny circular shape (indicated by black arrows, Figure 4D). This was not as prevalent in the WntA + PGE_2_ condition. However, the cells treated with WntA + PGE_2_ and Wort blocker, adopted the shiny circular phenotype seen in the WntA condition. Cells treated with WntA + PGE_2_ and H89 blocker adopted a circular appearance as well but a smaller population of these round cells were shiny.

Our experiments showed that cell viability was not affected but a distinct shiny circular cell appearance was observed, which is characteristic of a cell just prior to splitting into two daughter cells. Therefore, we also quantified the *split percentage*, defined as the percentage of cells that successfully divided into two daughter cells during the recorded time period. As expected, the NE-4C untreated cells demonstrated a split percentage of 100% (Figure 4C), indicating that all cells entering a mitotic phase resulted in cell division. A similar pattern was seen in PGE_2_-treated cells (97.5%). However, treatment of WntA resulted in a significant decrease of split percentage to 0% (*p* < 0.001), where mitotic cells appeared to become arrested in a round stage denoted in Figure 4D (*WntA* Image) with black arrows. The addition of 1 μM PGE_2_ to WntA-treated cells produced a significant increase in split percentage to 14.7% (*p* < 0.001, Figure 4C) as compared to WntA only treatment. The cells appear to resume their flat morphology. These results suggest that PGE_2_ treatment can modify Wnt-induced proliferation behaviour such as split percentage. Following treatment with either H89 or Wort, cells returned to a split percentage of 0% as seen with WntA only treatment (Figure 4C, D). This again indicates that PGE_2_ likely acts on the Wnt pathway through PKA and PI-3K to modify cell proliferation.

**Figure 5:**
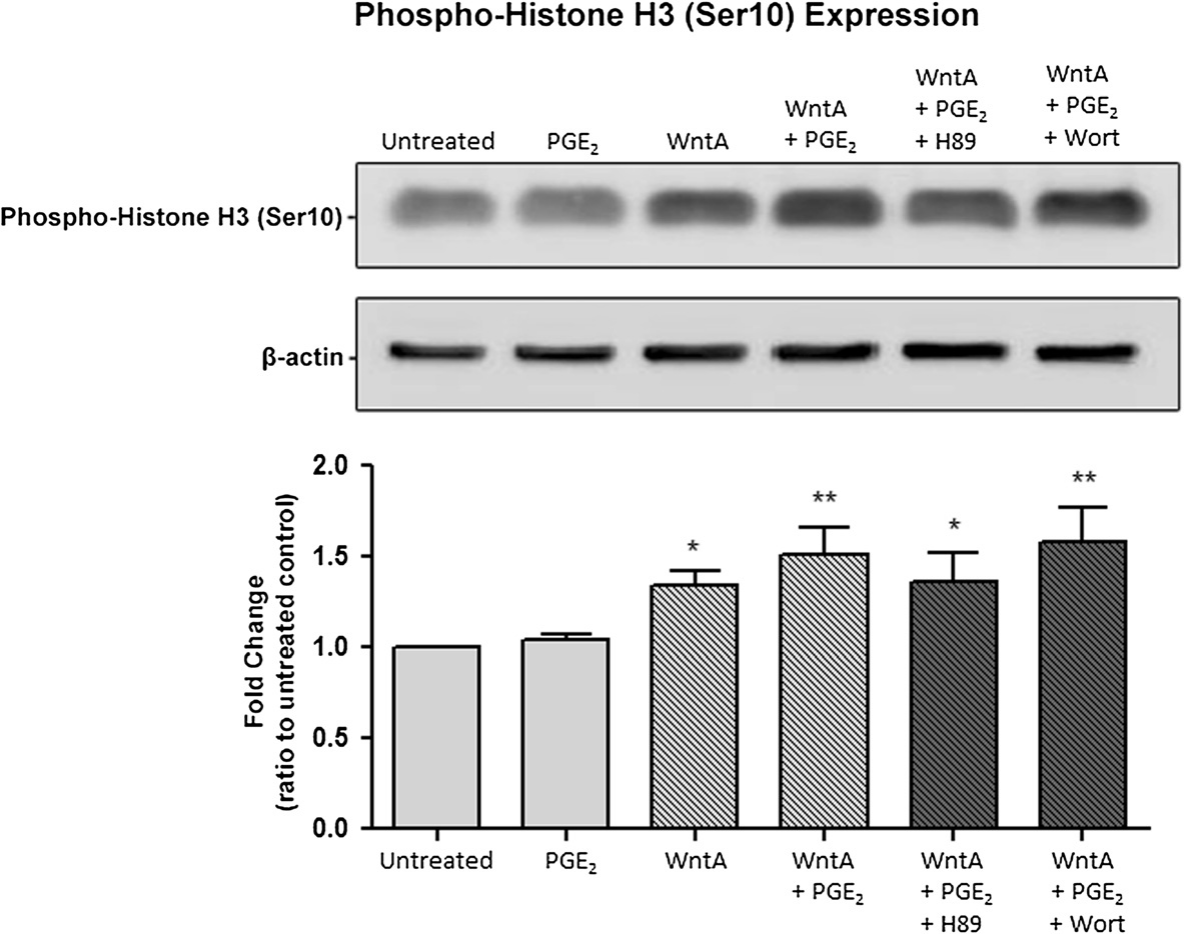
**PGE**_**2**_**-dependent effect on phospho-histone H3 (Ser10) expression.** Western blot analysis was used to determine Phospho-Histone H3 (Ser10) protein (17 kDa). The expression of Phospho-Histone H3 (Ser10) represented in fold change was 1, 1.04, 1.35, 1.52, 1.36, and 1.58, respectively. The error bars represent + SEM and values were considered significantly different from untreated *p < 0.05, **p < 0.01. Average measurements represent protein from three independent experiments (*N* = 3). β-Actin was used to indicate equal loading.

To further confirm our results of the cell splitting behaviour, we measured the level of Phospho-Histone H3 (Ser10) (Figure 5) since phosphorylation at Ser10 is tightly associated with chromosome condensation and segregation that occurs during mitosis [47-49]. Compared to untreated cells, PGE_2_ only-treated cells did not display a significant difference. However, when compared to untreated NE-4C cells, cells treated with WntA, WntA + PGE_2_ and WntA + PGE_2_ with H89 or Wort treatment led to a significance increase in Phospho-Histone H3 (Ser10) expression. RQ values were 1.35 (*p* = 0.033), 1.52 (*p* = 0.001), 1.36 (*p* = 0.027), and 1.58 (*p* = 0.005), respectively. This revealed that although cell numbers were lower under these conditions, the relative expression of Phospho-Histone H3 (Ser10) was significantly higher, indicating that a greater percentage of cells were undergoing mitosis when exposed to these treatments compared to untreated cells. This correlates with our finding that a larger proportion of cells under these conditions adopts and seems to be arrested in a round stage characteristic of cells undergoing mitosis.

### Prostaglandin E_2_ increases active β-catenin expression in Wnt-induced NE-4C cells

**Figure 6:**
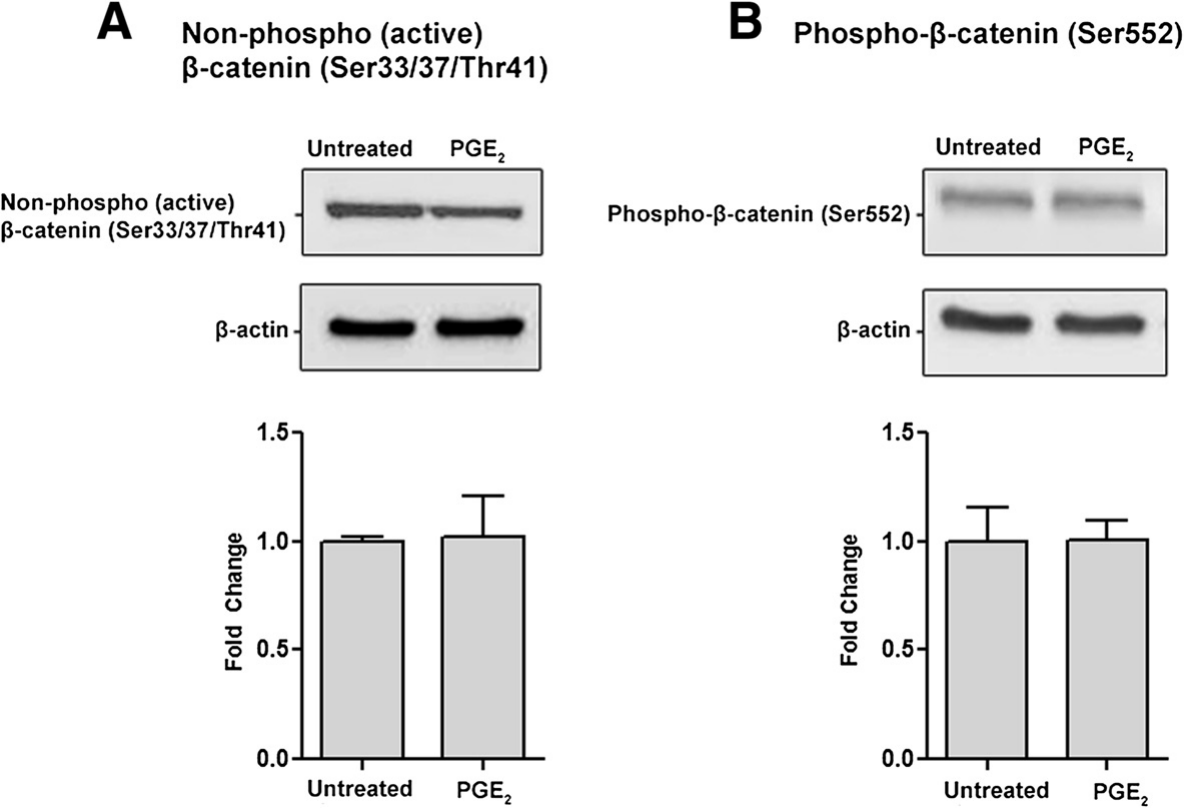
**PGE**_**2**_**-dependent effect on β-catenin expression in NE-4C cells.** Western blot analysis was used to determine two forms of active β-catenin: **(A)** non-phospho-(active) β-catenin (Ser33/37/Thr41) and **(B)** phospho-β-catenin (Ser552) (92 kDa). Addition of PGE_2_ to NE-4C cells did not yield a significant difference in levels of either active form of β-catenin compared to control. The error bars represent + SEM and values were considered significantly different from control at *p < 0.05, **p < 0.01. Average measurements represent protein from three independent experiments (*N* = 3). β-Actin was used to indicate equal loading.

**Figure 7:**
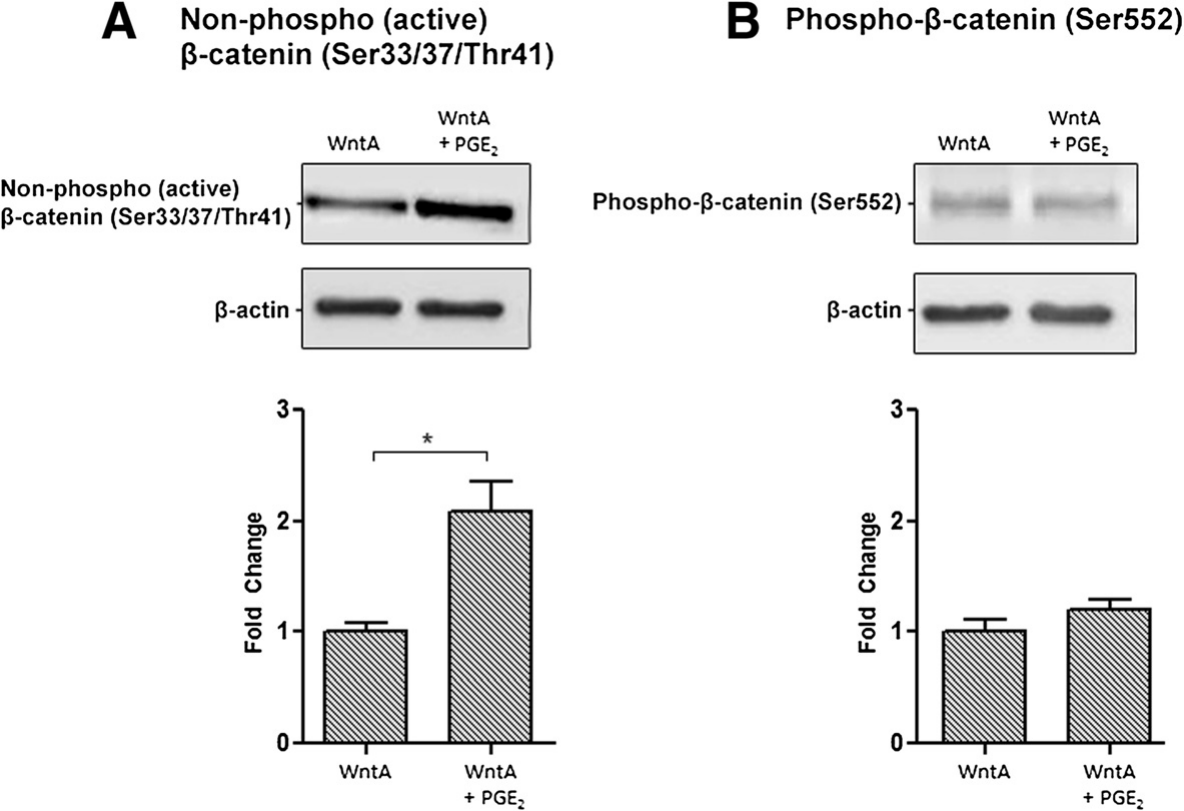
**PGE**_**2**_**-dependent effect on β-catenin expression in Wnt-activated NE-4C cells.** Western blot analysis was used to determine two forms of active β-catenin: non-phospho-(active) β-catenin (Ser33/37/Thr41) and phospho-β-catenin (Ser552) (92 kDa). **(A)** The expression of active β-catenin represented in fold change was 1, 2.09, 1.61, and 1.98, respectively. The error bars represent + SEM and values were considered significantly different from control at *p < 0.05. Only PGE_2_ + WntA condition was significantly different from WntA only condition. **(B)** There was no significant difference in phospho-β-catenin (Ser552) expression between the conditions. Average measurements represent protein from three independent experiments (*N* = 3). β-Actin was used to indicate equal loading.

β-catenin is a key effector in the canonical Wnt signalling pathway that regulates downstream gene transcription [50]. β-catenin levels can be intricately regulated at multiple phosphorylation sites. Phosphorylation at Ser33, Ser37, and Thr41 leads to its destabilization and primes it for degradation [51], while phosphorylation at Ser552 has been correlated with β-catenin nuclear accumulation [52,53]. We tested the levels of non-phospho-(the active form) β-catenin (Ser33/37/Thr41) and phospho-β-catenin (Ser552). The addition of PGE_2_ only to NE-4C cells did not significantly change the levels of either form of β-catenin (Figure 6A and B). However, adding PGE_2_ to WntA-induced NE-4C cells lead to a significant 2.1 fold increase in non-phospho-(active) β-catenin (Ser33/37/Thr41) levels compared to the WntA only treated condition (Figure 7A). There was no significant difference in Phospho-β-catenin (Ser552) levels between the sample conditions (Figure 7B), suggesting that phosphorylation of β-catenin at Ser552 is likely not involved with the behavioural differences in NE-4C cells described earlier. These results indicate that PGE_2_ may interact with the canonical Wnt signalling pathway by regulation of non-phospho-(active) β-catenin (Ser33/37/Thr41) levels.

### Prostaglandin E_2_ regulates expression of Wnt-target genes in Wnt-induced NE-4C cells

**Figure 8:**
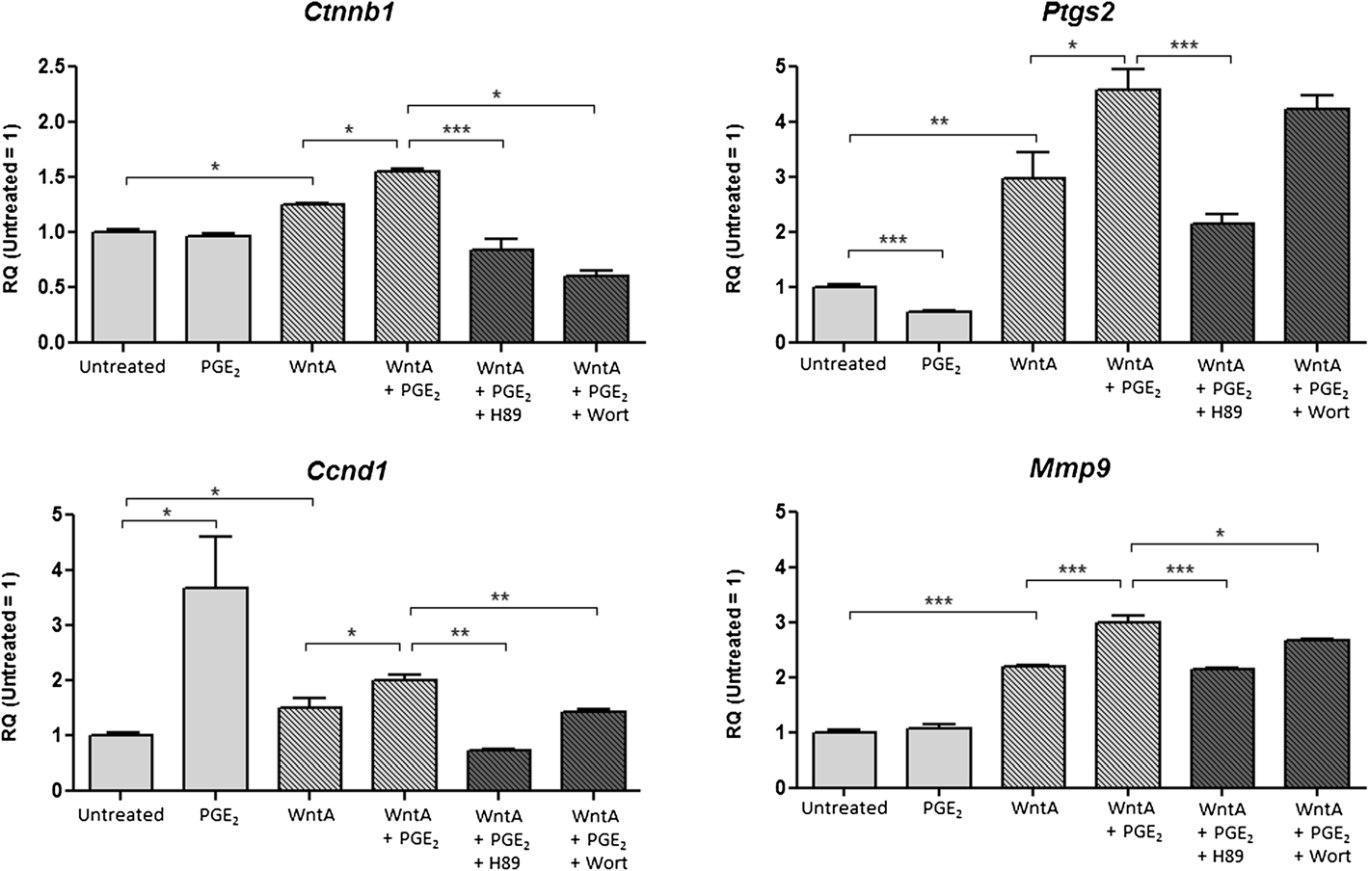
**PGE**_**2**_**-dependent effect on Wnt-target genes.** Real-time PCR was used to determine the RQ value for *Ctnnb1*, *Ptgs2*, *Ccnd1*, and *Mmp9*. The expression of *Ctnnb1* represented in fold change was 1, 0.97, 1.25, 1.55, 0.84, and 0.60, respectively. The fold change expression of *Ptgs2* was 1, 0.56, 2.99, 4.59, 2.16, and 4.22. The fold change expression of *Ccnd1* was 1, 3.68, 1.50, 1.99, 0.74, and 1.42. *Mmp9* fold change expression was 1, 1.08, 2.19, 3.00, 2.16, and 2.68, respectively. The error bars represent + SEM and values were considered significantly different from control at *p < 0.05, **p < 0.01, and ***p < 0.001. Average measurements are from three independent experiments (*N* = 3).

To investigate whether the addition of PGE_2_ can influence gene transcription relevant to the canonical Wnt pathway, we screened 29 target genes using Custom TaqMan® Array Plates. We found that *Ctnnb1*, *Ptgs2*, *Ccnd1*, and *Mmp9* were differentially regulated (data not shown). Their expression was confirmed with real-time PCR using RNA derived from the same treatment conditions used for behavioural analyses, which includes 1 μM PGE_2_, 2 μM Wnt Agonist (WntA), or 2 μM WntA with the addition of 1 μM PGE_2_. Kinase blockers (H89 or Wort) were added to PGE_2_ + WntA treated cells to determine the potential contribution of PKA and PI3K activity via PGE_2_ signalling. Our real-time PCR results indicate that PGE_2_ affects the expression levels of all Wnt-target genes tested (Figure 8).

*Ctnnb1* (beta-catenin) levels were not altered with the addition of PGE_2_ when compared to untreated NE-4C cells, but cells treated with WntA showed a significant increase of RQ value 1.25 (*p* = 0.0372). Addition of PGE_2_ to WntA-induced cells led to a further increase of *Ctnnb1* level to an RQ value of 1.55, which was significantly different from the WntA-only condition (*p* = 0.0131). This pattern was consistent with the expression of phospho (active) β-catenin (Ser33/37/Thr41) protein quantified earlier using Western blot analysis. Addition of H89 or Wort to PGE_2_ + WntA treated cells resulted in RQ values to 0.83 and 0.60, respectively, compared to untreated cells which was a significant decrease compared to the PGE_2_ + WntA condition (*p* > 0.001, *p* > 0.001). The PKA and PI3K blockers, H89 and Wort, appeared to remove the effect of PGE_2_ on *Ctnnb1* expression in WntA-induced cells, while also reversing the influence on *Ctnnb1* levels from WntA-only treatment. This suggests that PKA and PI3K signalling may modify *Ctnnb1* expression through PGE_2_ signalling.

NE-4C cells treated with PGE_2_ alone had a significant decrease in *Ptgs2* (prostaglandin-endoperoxide synthase 2; gene encoding COX-2) mRNA levels compared to untreated cells (RQ = 0.56, *p* < 0.001), while cells treated with WntA had a significant increase of RQ value 2.99 (*p* = 0.00286). In contrast, when PGE_2_ was added to WntA-induced NE-4C cells, *Ptgs2* expression was further elevated with an RQ value of 4.59 compared to untreated. This value was significantly different from the PGE_2_ + WntA condition (*p* = 0.015). Addition of H89 or Wort to PGE_2_ + WntA treated cells resulted in RQ values of 2.16 and 4.22, but only the H89 treatment was significantly different from the PGE_2_ + WntA condition (*p* < 0.001). This suggests that the effect of PGE_2_ on WntA-induced cells may be through PKA.

Expression of *Ccnd1* (cyclin D1) was also altered. Administration of PGE_2_ treatment to NE-4C cells correlated with a significant increase of an RQ value to 3.68 (*p* = 0.045) compared to untreated cells, while WntA-treated cells had a significant increase of RQ value to 1.50 (*p* = 0.048). Addition of PGE_2_ to WntA-activated cells was associated with a further increase of *Ccnd1* expression, with an RQ value 1.99 compared to untreated cells, which was significantly different from WntA-only treated cells (*p* = 0.047). H89 or Wort added to PGE_2_ + WntA treated cells had RQ values of 0.74 and 1.42, respectively, which was significantly different from the PGE_2_ + WntA condition (*p* = 0.0054, *p* = 0.0078). The blockers, H89 and Wort, seemed to attenuate the increase of *Ccnd1* levels associated with the addition of PGE_2_ to WntA-induced cells.

In comparison to untreated NE-4C cells, PGE_2_ treatment did not change levels of *Mmp9* (matrix metalloproteinase 9). However, when compared to WntA-induced NE-4C cells, addition of PGE_2_ treatment to WntA-treated cells caused a significant increase in expression level (*p* < 0.001). Specifically, with WntA treatment, *Mmp9* expression was significantly elevated to an RQ value of 2.19 (p < 0.001) compared to untreated cells, but addition of PGE_2_ to WntA-induced cells resulted in a further rise of *Mmp9* expression with an RQ value of 3.00. H89 and Wort were added to PGE_2_ + WntA treated cells and RQ values for *Mmp9* were 2.16 and 2.68, respectively, compared to the untreated condition. These values were significantly different from the PGE_2_ + WntA condition. This indicates that the use of H89 and Wort diminished the increase in *Mmp9* expression as a result of PGE_2_ treatment on WntA-induced cells.

Overall, these results demonstrate that PGE_2_ can raise the expression of Wnt-target genes, specifically, *Ctnnb1*, *Ptgs2*, *Ccnd1*, and *Mmp9*, in WntA-induced NE-4C cells. Since H89 and Wort attenuated the changes caused by PGE_2_, PKA and PI3K likely serve as a molecular link for the interaction between the PGE_2_ and canonical Wnt signalling pathways.

## Discussion

Cell migration and proliferation are crucial components of neural development. Previous studies have shown that elevated levels of PGE_2_ can result in increased cell motility and proliferation in various non-neuronal cells [46,54-56]. Recent evidence indicates that abnormalities in cell behaviour can result from the interaction between PGE_2_ with Wnt signalling pathways [44,57]. Our current study provides evidence, for the first time, for the cross-talk between these two pathways in neural stem cells. We report that PGE_2_ treatment elicits changes in cell behaviour such as an increase in components of cell motility and proliferation, as well as expression of Wnt-target genes, in Wnt-activated NE-4C stem cells. Moreover, the stimulatory effects of PGE_2_ are subdued through the inhibition of downstream pathway kinases, PKA and PI-3K, suggesting that PGE_2_ acts through these particular kinases to converge with the Wnt pathway.

Previous studies have shown that PGE_2_ can increase or decrease the activity of canonical Wnt signalling. PGE_2_ activates several components of the canonical Wnt pathway in colorectal cancer cells (reviewed in [42]). Specifically in these cells, PGE_2_ stimulated a significant increase in the activity of Wnt transcription factors, T cell factor-4 (Tcf-4), as well as elevated protein levels of Wnt-target genes [58]. PGE_2_ acted through its EP2 receptor to modulate β-catenin activity of the Wnt pathway, promoting the growth of colon cancer cells [44]. Wnt activation induced by PGE_2_ also contributed to abnormal proliferation resulting in enhanced gastric tumorigenesis [57]. Furthermore, PGE_2_–regulated Wnt signalling had a hepatoprotective effect, aiding in liver regeneration [59]. In pre-osteoblastic cells, concentration-dependent treatment of PGE_2_ modulated Wnt signalling by altering protein expression of pathway activators, β-catenin and low-density lipoprotein receptor-related protein 5/6 (LRP 5/6), as well as Wnt inhibitor, dickkopf-1 (DKK-1); low doses of PGE_2_ promoted the Wnt pathway while high doses inhibited it [37]. PGE_2_ also modified Tcf-luciferase activity of Wnt signalling through the same dose effect [37]. Additionally, in human colorectal adenoma and carcinoma cells, PGE_2_ treatment up-regulated the protein expression of the Wnt target gene, leucine-rich G-protein coupled receptor 5 (LGR5), which internalizes FZD co-receptor LRP6 and decreases Wnt activity [60]. Altogether, these studies reveal that the interaction between PGE_2_ and Wnt signalling can have different effects depending on the dose of PGE_2_ administered and the specific cell type.

We reveal that PGE_2_ increases the final distance and path length travelled, as well as the average speed of migration in Wnt-activated neuroectodermal stem NE-4C cells. We also show that PGE_2_ alters the phenotype of Wnt-treated cells, which corresponds to an increase in split percentage. Aberrations in cell motility and proliferation behaviour could have detrimental effects to early development of the nervous system. This is because proper neural development requires an orchestrated system of cellular events, such as migration and proliferation, to occur over particular windows of time [61]. Careful control of these crucial neurobiological processes during prenatal development is required for the formation of complex layered structures in the brain like the cerebral cortex, hippocampus, and cerebellum [62,63].

Our study adds to the current body of research by showing that PGE_2_ interferes with the Wnt pathway by attenuating Wnt-dependent cell behaviour in NE-4C cells. This is important because Wnt signalling is involved in a myriad of regulatory processes important for the development and organization of the nervous system [64]. It is thoroughly established that Wnt signalling is instrumental to normal anterior-posterior patterning of the embryo [65]. Wnt proteins are key regulators for the formation of the neural tube, as well as neuronal migration and differentiation [40,64]. Wnt signalling also modulates neurite outgrowth [66], axon growth and guidance [67-70], dendritic development and arborization [71,72], radial migration [73], and synapse formation and plasticity [74,75]. Moreover, Wnt signalling is crucial in neuronal fate determination, particularly in the specification and differentiation of neuronal precursors in the midbrain [76] and forebrain [77,78]. Furthermore, epithelial stem cells require Wnt/β-catenin signalling for proliferation and quiescent division [79] and the balance between re-entry and exit of the cell cycle can be altered by Wnt/β-catenin signalling [80]. Additionally, aberrant cortical progenitor cell proliferation patterns and defective hippocampus development can result due to abnormal Wnt signalling [81]. Interestingly, recent findings provide evidence that defective Wnt signalling could contribute to the pathogenesis of psychiatric disorders like schizophrenia and ASD [82-84]. Specifically, *Wnt2*, located in the putative speech and language region at chromosome 7q31-33, has been identified as a susceptibility gene for autism. [85,86]. Given the importance of Wnt signalling in prenatal development and the existing interaction between Wnt and PGE_2_ pathways in NE-4C stem cells, alterations in levels of PGE_2_ via endogenous and exogenous means may have profound effects on nervous system development.

In addition to quantifying cell behaviour, we also demonstrate that PGE_2_ can affect the expression of non-phospho (active) β-catenin (Ser33/37/Thr41). Wnt/β-catenin signalling occurs through a complex, highly regulated pathway that involves the phosphorylation of multiple sites on β-catenin, which may promote its degradation or activation and subsequent nuclear internalization. For instance, the phosphorylation of sites Ser33, 37, and Thr41 targets β-catenin for ubiquitination and proteasomal degradation [87,88]. Quantification of β-catenin that is non-phosphorylated at these sites has become a common measurement for active or stabilized β-catenin expression. Phosphorylation of β-catenin at the site Ser552 has also been correlated with increased β-catenin/TCF mediated transcriptional activity [89,90]. We found that PGE_2_ treatment administered to Wnt-activated cells increased the expression of non-phospho (active) β-catenin (Ser33/37/Thr41) protein. In contrast, the phospho- β-catenin (Ser552) levels remained unchanged. It has been established that the regulation of glycogen synthase kinase 3 beta (GSK3β) activity may control stabilization of β-catenin and increased levels of non-phospho (active) β-catenin (Ser33/37/Thr41) protein [91]. It is possible that PGE_2_ signalling may modify GSK3β activity but this remains to be determined. Nonetheless, the increased levels of non-phospho (active) β-catenin (Ser33/37/Thr41) quantified were in line with our gene expression results that also showed an increase in *Ctnnb1* expression as well as other Wnt-target genes. *Ctnnb1* encodes for the β-catenin protein, which can regulate cell growth and adhesion and is also a key downstream component of the canonical Wnt pathway. It has also been shown to regulate cortical size; enlarged cortices with increased cortical folds were observed in *Ctnnb1* transgenic mice [80]. Interestingly, brain overgrowth and abnormal excess in number of neurons was measured in children with autism [92]. Gene expression of *Ctnnb1* was altered in both young and adult autistic cases [93]. Furthermore, de novo mutations of this gene and its relevant network have been ranked significantly as potential autism candidate genes [94,95]. Within the canonical Wnt pathway, the β-catenin/TCF complex can promote the transcription of target genes including *Ptgs2*[96], *Ccnd1*[97,98], and *Mmp9*[99,100]. Expression of these genes was increased as an effect of elevated PGE_2_ signalling in our study, and interestingly, previous studies have reported a link between these genes and ASD as described below.

*Ptgs2*, also known as COX-2, is the key enzyme in prostaglandin biosynthesis, including the production of PGE_2_. COX-2 is a crucial mediator of inflammation and prostanoid signalling [101,102]. Polymorphism of *Ptgs2* has been associated with ASD [103]. A recent clinical study proved the efficacy of a COX-2 inhibitor drug, celecoxib, as an adjunctive therapy in the treatment of autism: the treatment was superior for treating irritability, social withdrawal, and stereotypy of children with autism [104].

Another gene affected was *Ccnd1*. This gene encodes for a protein in the cyclin family, which are important regulators in cell cycle progression, transcription, and neuronal function [105,106]. The increased levels of *Ccnd1*, as a result of added PGE_2_, may be involved with the altered proliferation behaviour visualized in this study. Aberrant *Ccnd1* levels have also been associated with ASD. In autistic rat pups (model encompassed administration of valproic acid), *Ccnd1* expression was atypical in the cerebellum compared to controls [107]. Another study showed that the dysregulation of *Ccnd1* lead to abnormal cell cycle and proliferation, neuronal and network excitability and behaviour, and revealed its potential link to human neuro-cardio-facial-cutaneous and related syndromes, which are associated with developmental abnormalities, cognitive deficits, and autism [108]. Diminished expression of 22q11 genes, which disrupts cortical neurogenesis and cell migration, led to alterations in *Ccnd1* levels [109]. The authors explain that a developmental disruption, as such, may alter cortical circuitry and establish vulnerability for developmental disorders, including schizophrenia and autism.

*Mmp9* is a membrane of the matrix metalloproteinase (MMP) family, which can target many extracellular proteins including proteases, growth factors, and adhesion molecules [110] and are involved with the breakdown of the extracellular matrix in normal physiological processes such as embryonic development and tissue remodelling [111]. MMPs are also important in neuronal development, plasticity, and maintenance of neuronal health [112]. Mmp9 has also been shown to regulate the proliferation and migration of embryonic neural stem cells [99] and participate in neuronal differentiation by regulating neurite elongation and neuronal cell migration [113-115]. Therefore, altered *Mmp9* expression may contribute to the deviant behaviour observed in our study. *Mmp9* has also been associated with ASD [116]. Elevated levels of MMP9 protein were found in the amniotic fluid of ASD cases compared to controls [117]. Findings from this study provide evidence that molecular and physiological abnormalities in ASD may begin prenatally. *Mmp9* has also been implicated in Fragile X syndrome (FXS) [118], which is characterized by behaviours at the extreme of the autistic spectrum. Using in a mouse model of fragile x (Fmr1 KO), levels of MMP9 was found to be elevated in the hippocampus of Fmr1 KO mice [119]. Furthermore, Minocycline, a drug that inhibits MMP9 activity, has been shown to promote dendrite spine maturation and improve behavioural performance in Fmr1 KO mice [119]. These researchers continued their work in human trials and found that Minocycline taken as a daily dose for 8 weeks led to behavioural improvements in FXS patients. This was consistent with their fmr1 KO mouse model results, indicating that MMP9 activity alters underlying neural defects that contribute to behavioural abnormalities seen in ASD [120].

Taken altogether, our gene expression results not only show a potential interaction of the PGE_2_ and canonical Wnt pathway in the nervous system, but also provide further evidence for a link to ASD.

**Figure 9:**
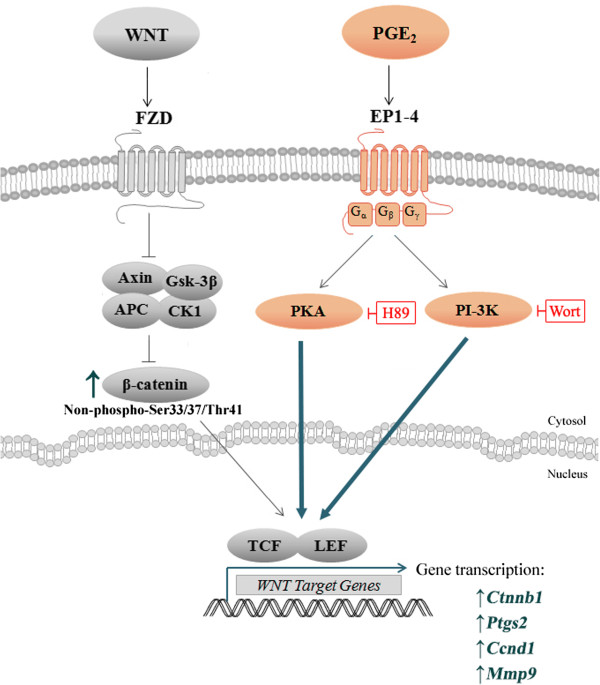
**A proposed model for PGE**_**2**_**-Wnt interactions in Wnt-induced NE-4C cells.** From the compilation of our results (bolded) and other studies, a schematic model is drawn of the mechanism by which PGE_2_ might interact with the canonical Wnt pathway.

We show that PGE_2_ interacts with canonical Wnt signalling through PKA and PI-3K to produce the reported behavioural changes in cell motility and proliferation, as well as gene expression. Specifically, we found that inhibiting these PGE_2_ downstream pathway kinases, PKA and PI-3K with H89 and Wort respectively, reduced the effect of PGE_2_. This is in line with previous literature, which found that the convergence of PGE_2_-dependent effects and the Wnt pathway can occur through the stimulation of PKA or PI-3K in embryonic kidney cells and colon cancer cells [44-46]. Moreover, similar stimulatory effects on cell migration induced by PGE_2_ in Wnt-activated NE-4C cells from our study were also exhibited in prostate cancer cells through the activation of PI-3K [121]. Our results revealed that H89 had a stronger effect than Wort, suggesting that PGE_2_ may predominately act through PKA; but future studies are needed to determine which EP receptors are involved. A proposed model is provided in Figure 9.

Increasing evidence for the contribution of environmental factors in the etiology of neurodevelopmental disorders like ASD has prompted urgency to reveal their potential exogenous causes and underlying mechanisms [122]. Environmental factors like exposure to drugs, toxins or infectious agents cause disruptions in PGE_2_ signalling by increasing the levels of oxidative stress, consequent lipid peroxidation, and the immunological response; these factors and consequences that disturb normal PGE_2_ signalling have all been linked to ASD [123]. We show that increased PGE_2_ signalling can modify cell migration, proliferation behaviour, and gene expression in Wnt-activated NE-4C stem cells. Aberrant cell migration and proliferation are pathophysiologic mechanisms that impact the brain broadly, and could be possible factors that contribute to the origination of neurodevelopment disorders. Abnormalities in structure, organization, and connectivity of the brain are all indicators of irregular neural cell migration and proliferation. Local distortions in neural cytoarchitecture, dysplasia, and hypoplasia have been described in brains of autistic subjects [124]. Moreover, structural abnormalities and atypical connectivity of the brain in ASD has been identified by a number of research groups [123,125-128]. Noteworthy, areas of the brain that would be most impacted by dysregulation in neuronal migration and proliferation—that is the cerebellum, cerebral cortex, and hippocampus—are also implicated in ASD [124,129-131]. Despite the assumptions that can be made from our in vitro results, in vivo models must be employed to further describe the possible effects of PGE_2_ and its interaction with morphogenic signalling pathways, such as Wnt, during prenatal development.

## Conclusions

PGE_2_ is an important bioactive lipid signalling molecule and its interaction with Wnt signalling pathway could have significant effects on prenatal development. Our study shows for the first time that PGE_2_ can affect Wnt-dependent cell behaviours and gene expression in neuroectodermal stem cells through PKA and PI-3K. Aberrant PGE_2_ and Wnt signalling have been linked to ASD. Moreover, altered migration and proliferation due to irregular gene expression during embryonic development in ASD have been suggested in previous studies. Our *in vitro* study provided further evidence that these aberrations may be potential mechanisms in the genesis of neurodevelopment disorders like ASD.

## Methods

### Cell culture

Mouse NE-4C cells were obtained from American Tissue Culture Collection (ATCC) and grown in Minimum Essential Medium (MEM) supplemented with 10% fetal bovine serum, 2 mM glutamine, 1 X penicillin-streptomycin mixture (Invitrogen). Cells were maintained in an incubator containing 5% CO_2_ at 95% humidity 37°C. Cells were plated on 0.01% poly-L-lysine (Sigma) coated 100 mm culture plates (BD Falcon) and were subcultured at a 1:10 ratio. Supplemented MEM was changed every 2–3 days.

### Cell culture-treatments

NE-4C cells (ATCC) were dissociated with 0.05% trypsin-EDTA, pelleted and resuspended in complete medium as described above. The cells were plated on poly-L-lysine 0.01% (Sigma, MW 70000–150000 kDa) on 35 mm tissue culture dishes (Sarstedt). Plated cells were incubated in 5% CO_2_ at 95% humidity 37°C overnight before treatment with Wnt Agonist (WntA, Calbiochem), prostaglandin E_2_ (PGE_2_, Sigma) and/or blockers. WntA (2 μM), PGE_2_ (1 μM), H89 dihydrochloride hydrate (H89, 10 μM, Sigma), Wortmannin (WORT, 1 μM, Sigma) or an equivalent volume of vehicle were added to each well. Cells were treated for 24 hours.

### Reverse transcription and real-time PCR

Total RNA was extracted from NE-4C cells using the NucleoSpin RNA/Protein Kit (Macherey-Nagel) and was reverse-transcribed into cDNA using MMuLV reverse transcriptase (New England Biolabs) according to manufacturer’s instructions. Primer3 Input software (v. 0.4.0) was used to design forward and reverse primers for EP receptors and have been previously noted [12]. Selection of Wnt-target genes was determined using Custom TaqMan® Array Plates (Life Technologies) as a screening tool (data not shown). Genes that had a greater than 1.8 fold-change were selected for further validation and forward and reverse primers were designed (Table 1). Real-time PCR was performed using the 7500 Fast Real-time PCR system (Applied Biosystems) and the ΔΔC_T_ method was applied to calculate the expression of transcripts. Hypoxanthine phosphoribosyl transferase (HPRT) and Phosphoglycerate Kinase 1 (PGK1) served as endogenous controls. The relative quantification (RQ) ratios were determined from the average of three technical replicates from three biological replicates.

**Table 1:** Forward and reverse primer sequences for real-time-PCR

**Gene**	**Primer sequences *(5′ *** **→ 3′)**	**Amplicon size (base pair)**
*Hprt*	F: TCCATTCCTATGACTGTAGATTTTATCAG	*75*
	R: AACTTTTATGTCCCCCGTTGACT	
*Pgkl*	F: CAGTTGCTGCTGAACTCAAATCTC	*65*
	R: GCCCACACAATCCTTCAAGAA	
*Ptgs2*	F: CAGCCAGGCAGCAAATCC	81
	R: TTATACTGGTCAAATCCTGTGCTCAT	
*Ctnnbl*	F: GGACGTTCACAACCGGATTG	71
	R: GAGAATAAAGCAACTG CACAAACAA	
*Ccndl*	F: GCACTTTCTTTCCAGAGTCATCAA	79
	R: CTCCAGAAGGGCTTCAATCTGT	
*Mmp9*	F: TCGCGTGGATAAGGAGTTCTCT	73
	R: ATAGGCTTTGTCTTGGTACTGGAAGA	

### Western blot analysis

Total protein was extracted from NE-4C cells using the NucleoSpin RNA/Protein Kit (Macherey-Nagel). Samples were separated by polyacrylamide gel electrophoresis (PAGE). Primary antibodies used for EP expression levels include rabbit polyclonal anti-EP1, −EP2, −EP3, −EP4 (1:200; Santa Cruz Biotechnology). Detection of rabbit monoclonal anti-Phospho-Histone H3 (Ser10) (1:1000; Cell Signaling) was used as a measure of cell splitting behaviour. Primary antibodies used for β-catenin expression levels were rabbit monoclonal anti-non-phospho (Active) β-catenin (Ser33/37/Thr41) and rabbit polyclonal anti-phospho-β-catenin (Ser552) (1:1000; Cell Signaling). Blots were reprobed with mouse monoclonal anti-*β*-Actin (1:10,000; Abcam). Visualization of bound anti-rabbit and anti-mouse horseradish peroxidise-conjugated secondary antibodies was achieved by incubation with ECL Prime Western Blotting Detection Reagent (GE Healthcare) and detection by Geliance 600 Imaging System (Perkin Elmer).

### Immunocytochemistry

NE-4C cells were seeded onto 35 mm culture plates containing poly-L-lysine coated coverslips and grown overnight at 37°C. The cells were fixed with 50% acetone and 50% methanol for 20 minutes at −20°C and washed with phosphate buffered saline (4.3 mM Na_2_HPO_4_, 137 mM NaCl, 2.7 mM KCl, 1.4 mM KH_2_PO_4_). Cells were then incubated with primary antibodies in PBS with 0.3% Triton-X 100 and 2% Normal Goat Serum. Cellular localization of the EP receptors was determined by incubation with anti-EP primary antibodies as described above along with mouse monoclonal anti-Lamin A + C nuclear envelope marker (1:200; Abcam), anti-58 K Golgi marker [58 K-9] (1:100; Abcam), anti-PDI endoplasmic reticulum marker [RL90] (1:100; Abcam) or *β*-Actin (1:1000; Abcam) at room temperature for 1 hour. Following primary antibody incubation, cells were washed three times with PBS-T for 15 min and incubated with secondary antibodies in PBS-T and 2% NGS for 1 hour at room temperature in the dark. Secondary antibodies used were anti-rabbit fluorescein isothiocyanate (FITC) (1:100; Jackson ImmunoResearch Laboratories) and anti-mouse Texas Red (1:200; Jackson ImmunoResearch Laboratories). Cells were then washed twice with PBS-T for 10 min, followed by a 20 minute incubation of 4′,6-diamidino-2-phenylindole (DAPI) (1:2000; Molecular Probes) at room temperature. Cells were washed twice with PBS-T for 5 min and coverslips were mounted on glass microscope slides with mounting media (Vectashield). The staining was visualized and captured using an Eclipse 80i upright fluorescent microscope with DS-5MC camera (Nikon).

### Time-lapse imaging and analysis

Cell behaviour was recorded using Nikon Eclipse Ti-E microscope. Three biological replicates of each treatment condition were performed (*N = 3*), where an average of 150 cells were tracked. Micrographs were automatically captured every 10 minutes for a 24 hour period from a minimum of three fields. To maintain conditions physiologically suitable for the cells, an enclosed chamber was mounted to the microscope, which was equipped with CO_2_ supply and temperature thermostat. Cells were kept at 5% CO_2_, 95% humidity, 37°C. Measurements were completed using NIS Elements software, including a specialized tracking module. Final distance from origin, path length, and average speed were tracked and calculated from an average of 150 cells per treatment condition. Initial and final cell counts were used to determine fold change as a measurement of proliferation. Split percentage was quantified as a measurement of proliferation behaviour. Split percentage was defined as the percentage of cells that fulfilled the complete cell cycle, which was evaluated based on whether the parent cell could successfully split into two daughter cells.

### Cell viability analysis

Cells were disassociated and diluted with equal volumes of trypan blue dye (4%). Cell count averages were taken from a minimum of four hemacytometer squares to determine cell number and viability.

### Statistical analysis

All numerical data were presented as mean + SEM of three individual experiments. Statistical analysis was performed using student t-test or one-way analysis of variance (ANOVA) followed by Tukey post-hoc comparisons or Dunnett t-test (2-sided). Differences were considered statistically significant at *p < 0.05, **p < 0.01, or ***p < 0.001.

## Abbreviations

ASD: Autism spectrum disorders; COX-1,-2: Cyclooxygenases −1 and −2; Ccnd1: Cyclin D1; Ctnnb1: Beta-catenin; EP1-4: E-prostanoid 1 through 4; H89: Dihydrochloride hydrate; Mmp9: Matrix metalloproteinase 9; NE-4C stem cell: Neuroectrodermal stem cells; PGE2: Prostaglandin E2; PI-3K: Phosphatidylinositide 3-kinases; PKA: Protein kinase A; PKC: Protein kinase C; Ptgs2: Prostaglandin-endoperoxide synthase 2; Wnt: Wingless-related MMTV integration site; WntA: Wnt agonist; Wort: Wortmannin.

## Competing interests

The authors declare that they have no competing interests.

## Authors’ contributions

CTW designed and performed experiments, acquisition and analysis of data, and wrote the manuscript. EA performed experiments and acquisition of data. HYL assisted with research and discussions. DAC participated in the design and coordination of the study, and was involved in drafting the manuscript. All authors read and approved the final manuscript.
